# Simultaneous intracranial and extracranial vertebral artery dissections: a case report

**DOI:** 10.1016/j.radcr.2023.05.002

**Published:** 2023-05-24

**Authors:** Hideki Endo, Hidetoshi Ono, Megumi Matsuda, Kenji Kamiyama, Hirohiko Nakamura

**Affiliations:** a Department of Neurosurgery, Nakamura Memorial Hospital, South 1, West 14, Chuo-ku, Sapporo, Hokkaido 060-8570, Japan; b Department of Radiology, Nakamura Memorial Hospital, South 1, West 14, Chuo-ku, Sapporo, Hokkaido 060-8570, Japan

**Keywords:** Computed tomography angiography, Headache, Magnetic resonance angiography, Magnetic resonance imaging, Vertebral artery, Vertebral artery dissection

## Abstract

Vertebral artery dissection can occur in intracranial or extracranial vertebral arteries. However, the simultaneous dissection of both intracranial and extracranial vertebral arteries is extremely rare. We describe a 45-year-old man with simultaneous intracranial and extracranial vertebral artery dissections in separate sites. The patient visited a neurosurgical clinic because of headache; he was diagnosed with right vertebral artery dissection and referred to our hospital. Magnetic resonance imaging showed an intramural hematoma and mild dilation of the external lumen in the right vertebral artery distal to the posterior inferior cerebellar artery. Magnetic resonance angiography revealed poor delineation of the entire right vertebral artery, including the proximal portion from the posterior inferior cerebellar artery. Computed tomography angiography revealed right extracranial vertebral artery dissection. Careful imaging assessment is thus important for identifying simultaneous intracranial and extracranial vertebral artery dissections.

## Introduction

Vertebral artery dissection (VAD) causes headache and neck pain and is one of the most important causes of stroke, sometimes resulting in life-threatening subarachnoid hemorrhage (SAH) [1]. VADs can occur in intracranial or extracranial vertebral arteries (VAs); however, to our knowledge, there are few reports of VADs developing simultaneously in both intracranial and extracranial VAs [2]. Herein, we report a case of simultaneous intracranial and extracranial VADs presenting headache and neck pain.

## Case report

**Fig. 1:**
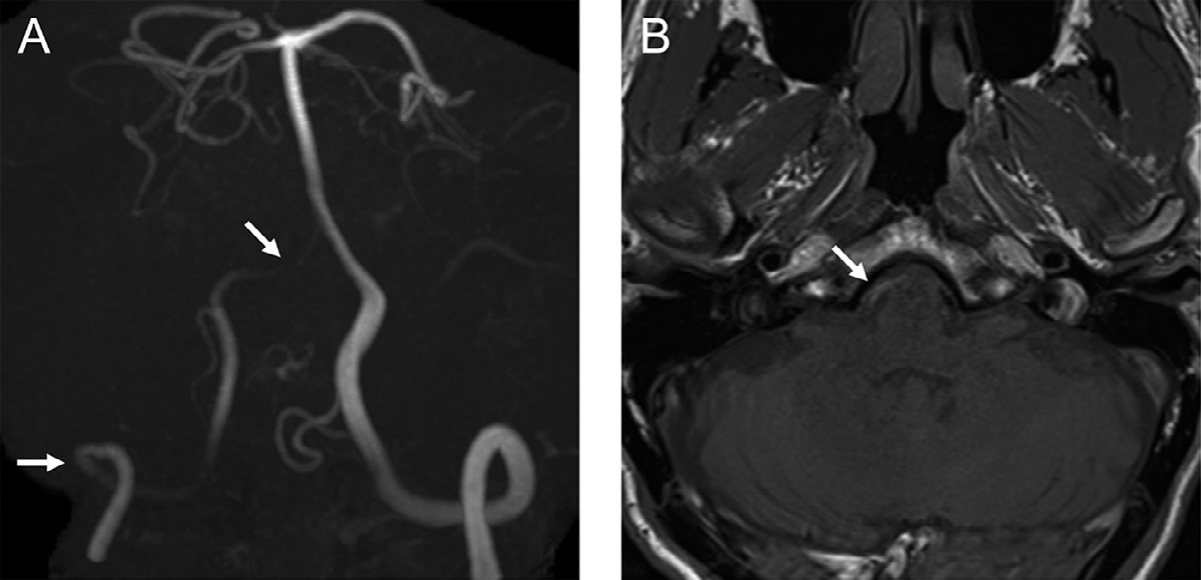
Intracranial vertebral artery dissection diagnosed by magnetic resonance angiography and magnetic resonance imaging. Magnetic resonance angiography showing the poor delineation of the entire right vertebral artery (arrows) (A). T1-weighted image demonstrating an intramural hematoma in the right intracranial vertebral artery distal to the posterior inferior cerebellar artery (arrow) (B).

**Fig. 2:**
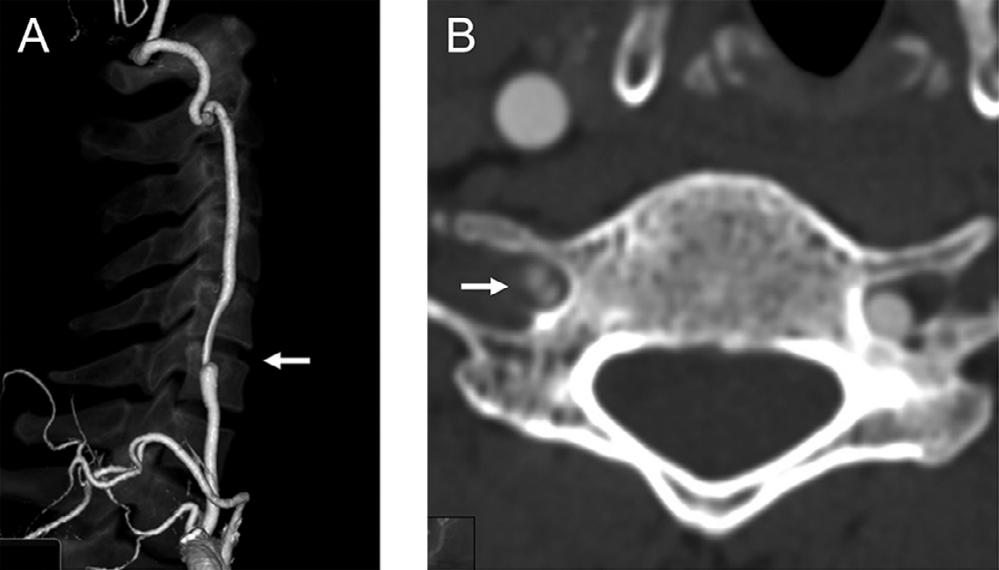
Extracranial vertebral artery dissection diagnosed by computed tomography angiography. Computed tomography angiography revealing the right extracranial vertebral artery dissection (arrow) (A). Axial image of computed tomography angiography demonstrating an intimal flap in the right extracranial vertebral artery (arrow) (B).

**Fig. 3:**
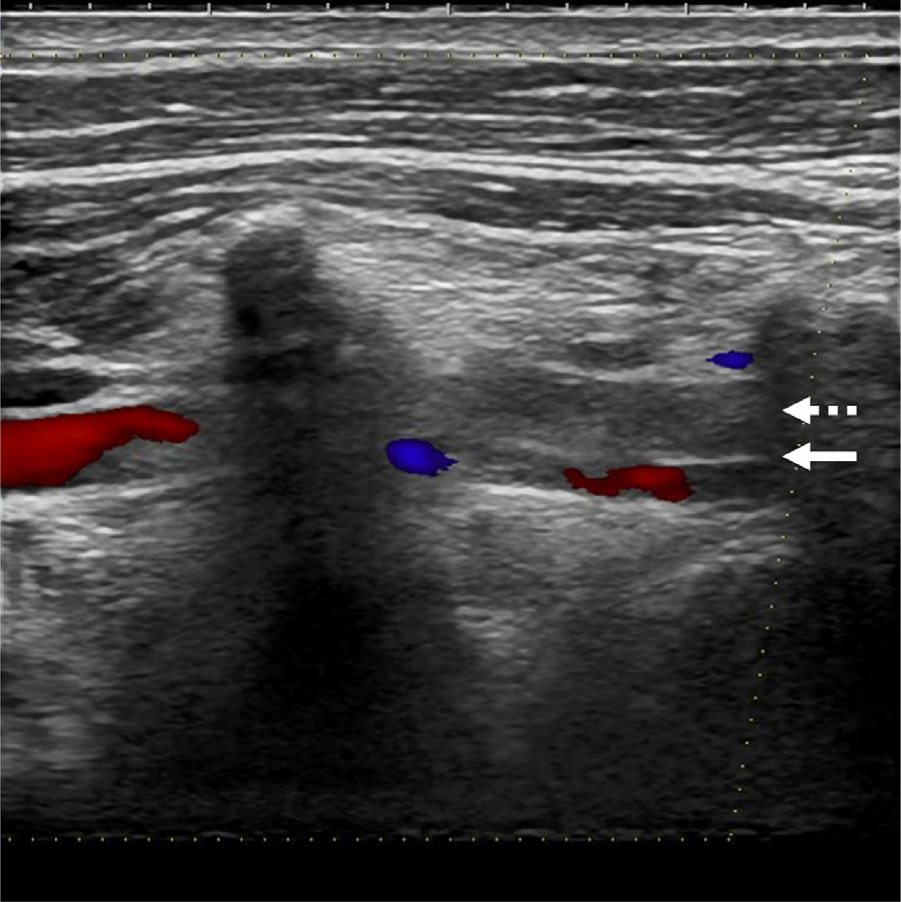
Extracranial vertebral artery dissection diagnosed by ultrasonography. Ultrasonography demonstrating an intimal flap (arrow) and decreased blood flow signal within the right extracranial vertebral artery. The dotted arrow indicates an intramural hematoma.

A 45-year-old man with no relevant medical history visited a neurosurgical clinic because of severe occipital headache with sudden onset 2 days earlier. He underwent magnetic resonance imaging (MRI) and magnetic resonance angiography (MRA) at the clinic. Although MRI excluded any intracranial hemorrhage, MRA revealed impaired delineation of the right VA, which had shown no abnormal findings in a scan 4 years earlier. The patient was diagnosed with right VAD and referred to our hospital for inpatient care. He had no apparent significant findings on physical examination other than headache and neck pain. He also had no atherosclerotic risk factors and no history of recent trauma. MRI/MRA (3 T Magnetom Skyra, Siemens, Erlangen, Germany) performed at our institution confirmed luminal narrowing of the entire right VA (Fig. 1A). Additionally, an intramural hematoma, mild stenotic changes, and mild dilation of the external lumen were observed in the right VA distal to the posterior inferior cerebellar artery (PICA) (Fig. 1B). These findings did not extend to the PICA origin. Computed tomography angiography (CTA) showed mild stenosis in the right VA distal to the PICA; the proximal intracranial VA, including the PICA origin, was intact. Furthermore, CTA revealed an intimal flap and stenosis at the level of the sixth cervical transverse foramen entrance of the right extracranial VA (Fig. 2). Ultrasonography demonstrated an intimal flap and decreased blood flow signal within the right extracranial VA (Fig. 3). We therefore diagnosed simultaneous intracranial (distal to the PICA portion) and extracranial VADs. We treated the patient conservatively—primarily with blood pressure and pain control—and he was discharged without any neurological deficits. His blood pressure remained stable, never elevated without antihypertensive medications. He was treated with indomethacin farnesil 220 mg/d for 2 weeks and thereafter did not require any pain management medications. Follow-up MRA 2 weeks after onset demonstrated improvement of poor right VA delineation.

## Discussion

We present a rare case of intracranial and extracranial VADs occurring simultaneously at separate sites. Although previous studies have described differences in clinical and radiological findings between intracranial and extracranial VADs [3,4], such studies did not report simultaneous intracranial and extracranial VADs despite the involvement of large numbers of patients. Some studies have reported extracranial VADs that extend intracranially as intracranial and extracranial VADs [5]; however, to our knowledge, there are few reports of intracranial and extracranial VADs occurring simultaneously at separate sites [2].

Partly because of the small numbers of reports [2,[6], [7], [8], [9], [10], [11], [12], [13], [14]], little is known about the clinical features of VADs that occur simultaneously in separate intracranial and extracranial VAs. A previous study reviewed 11 patients with simultaneous VADs of intracranial and extracranial portions [2], of whom 7 presented with cerebral ischemia and 4 with SAH; no cases were reported with headache only, as in the present case. These results suggest that simultaneous intracranial and extracranial VADs presenting with headache or neck pain only are very rare. While this may be true, the following reasons may also explain the very low recorded incidence of simultaneous intracranial and extracranial VADs presenting with headache only: first, no further examination may be performed in patients without cerebral ischemia or SAH; second, a diagnosis of VAD may not be reached in such patients; and third, such patients may not visit a hospital. Either way, accurate diagnosis is important because VAD can cause not only headache and neck pain, but also potentially stroke (including fatal SAH) [1].

In the present case, the initial MRA findings were useful for diagnosis (Fig. 1). Based on the MRI findings (Fig. 1B), we initially thought that our patient had only an intracranial VAD, but this diagnosis did not explain the poor delineation of the entire right VA, including the proximal portion from the PICA, on MRA (Fig. 1A). In particular, if the dissection had not extended to the ipsilateral PICA, the delineation from the proximal VA to the PICA should have been normal; this was not true in our case. These findings allowed us to make an accurate diagnosis using additional CTA and ultrasonography (Figs. 2 and 3). Note, however, that the aforementioned diagnosis does not apply if the intracranial VAD involves the PICA.

## Conclusion

We describe a rare case of intracranial and extracranial VADs occurring simultaneously in separate sites. The simultaneous occurrence of both intracranial and extracranial VADs is extremely rare but can occur. Careful imaging assessment is important for identifying simultaneous intracranial and extracranial VADs. The poor delineation on MRA of the entire VA, despite appearing intact up to the origin of the PICA, was useful in the diagnosis of this case.

## Authors’ contributions

**Hideki Endo:** Conceptualization, Methodology, Validation, Formal analysis, Investigation, Resources, Data curation, Writing - original draft, Writing - review & editing, Visualization, Project administration. **Hidetoshi Ono:** Validation, Formal analysis, Data curation, Writing - review & editing. **Megumi Matsuda:** Validation, Formal analysis, Writing - review & editing. **Kenji Kamiyama:** Validation, Writing - review & editing. **Hirohiko Nakamura:** Supervision.

## Patient consent

This study was approved by the institutional review board, and informed consent was obtained from the patient.

## Appendix Supplementary materials

Image, application 1

## References

[1] Debette S, Compter A, Labeyrie MA, Uyttenboogaart M, Metso TM, Majersik JJ (2015). Epidemiology, pathophysiology, diagnosis, and management of intracranial artery dissection. Lancet Neurol.

[2] Komiyama M, Ishiguro T, Matsusaka Y, Yasui T (2002). Simultaneous dissection of intra- and extracranial vertebral artery, report of two cases and review of literature. Acta Neurochir (Wien).

[3] Shin DH, Hong JM, Lee JS, Nasim R, Sohn SI, Kim SJ (2014). Comparison of potential risks between intracranial and extracranial vertebral artery dissections. Eur Neurol.

[4] Kobayashi H, Morishita T, Ogata T, Matsumoto J, Okawa M, Higashi T (2016). Extracranial and intracranial vertebral artery dissections: a comparison of clinical findings. J Neurol Sci.

[5] Di Meglio L, Mazighi M, Reiner P, Peres R, Guichard JP, Labeyrie MA (2019). Intracranial extension of extracranial vertebral dissection is associated with an increased risk of ischemic events. Stroke.

[6] Berger MS, Wilson CB (1984). Intracranial dissecting aneurysms of the posterior circulation. Report of six cases and review of the literature. J Neurosurg.

[7] Chiras J, Marciano S, Vega Molina J, Touboul J, Poirier B, Bories J (1985). Spontaneous dissecting aneurysm of the extracranial vertebral artery (20 cases). Neuroradiology.

[8] Hart RG, Easton JD (1985). Dissections. Stroke.

[9] Caplan LR, Zarins CK, Hemmati M (1985). Spontaneous dissection of the extracranial vertebral arteries. Stroke.

[10] Mas JL, Bousser MG, Hasboun D, Laplane D (1987). Extracranial vertebral artery dissections: a review of 13 cases. Stroke.

[11] Youl BD, Coutellier A, Dubois B, Leger JM, Bousser MG (1990). Three cases of spontaneous extracranial vertebral artery dissection. Stroke.

[12] Halbach VV, Higashida RT, Dowd CF, Fraser KW, Smith TP, Teitelbaum GP (1993). Endovascular treatment of vertebral artery dissections and pseudoaneurysms. J Neurosurg.

[13] McCormick GF, Halbach VV (1993). Recurrent ischemic events in two patients with painless vertebral artery dissection. Stroke.

[14] Mascalchi M, Bianchi MC, Mangiafico S, Ferrito G, Puglioli M, Marin E (1997). MRI and MR angiography of vertebral artery dissection. Neuroradiology.

