# Supplementary material for: Simultaneous intracranial and extracranial vertebral artery dissections: a case report
Source: Radiol Case Rep. 2023 May 24;18(8):2590–3. doi: 10.1016/j.radcr.2023.05.002 (PMC10232462; doi:10.1016/j.radcr.2023.05.002)
Supplement: Supplementary file 1 [file mmc1.docx]

**Highlights**

- Vertebral artery dissection (VAD) can occur in intracranial or extracranial VAs
- We report a rare case of simultaneous VADs of intracranial and extracranial VAs
- Intracranial VAD with impaired delineation of the entire VA was detected
- Computed tomography angiography revealed extracranial VAD
- Careful imaging assessment is important for identifying simultaneous VADs
